# Application of solar-based oxidation to the management of empty pesticide container rinse water in Bolivia

**DOI:** 10.12688/openreseurope.13555.1

**Published:** 2021-06-18

**Authors:** Raquel Mencia Torrico, María del Mar Micó Reche, Alberto Cruz Alcalde, Rossmary Violette Romero Olarte, Henry Antezana Fernández, María Mercedes Álvarez Caero, Carmen Sans Mazón

**Affiliations:** 1 Water and Environmental Sanitation Center, C.A.S.A.-FCyT, Universidad Mayor de San Simón (UMSS), C/Sucre y parque La Torre, Cochabamba, 5783, Bolivia; 2 Chemical Engineering and Analytical Chemistry, University of Barcelona, C/Martí i Franquès 1, Barcelona, 08028, Spain

**Keywords:** pesticides, wastewater, solar driven processes, photo-Fenton, empty containers, pollution, atrazine, dimethoate

## Abstract

**Background: **The management of empty pesticide containers (EPC) in Bolivia has been recently promoted as a control strategy for dispersed pollution in surface and underground water bodies, as well as in soil. It comprises the rinsing and proper disposal or reuse of clean EPC. However, the rinsing transfers the hazard to water, which must be properly treated before being discharged.

**Methods: **In this study, solar photo-Fenton at low Fe ^2+^ doses were tested at pilot plant scale in Cochabamba (Bolivia) for the removal of pesticides dimethoate and atrazine in their commercial form, spiked in river water.

**Results: **The results demonstrated that solar photo-Fenton ([H _2_O _2_] _0_ = 200 mg L ^-1^) with Fe ^2+^ concentrations between 0.5 and 1.5 mg L ^-1 ^is an effective method to remove dimethoate and atrazine, at an initial concentration of 10 mg L ^-1 ^each. Efficiency increased when increasing Fe ^2+^ doses, achieving a removal over 99% of both pesticides after a solar irradiation period of 60 minutes (corresponding to an accumulated energy of 4.96 kJ L ^-1^).

**Conclusions: **The presence of high concentrations of natural components of river water, mainly organic and inorganic carbon species, would have contributed to hydroxyl radical scavenging, explaining, together with the low iron dose applied, the high energy (irradiation time) and high hydrogen peroxide concentration required to produce pesticide depletion. Additionally, the relatively low oxidant consumption and mineralization observed leave room for process optimization regarding oxidant and catalyst doses and warrant further studies on its coupling with biological or other post-treatments for the removal of transformation products.

## Plain language summary

The management of empty pesticide containers (EPC) in Bolivia has recently been promoted as a control strategy for dispersed pollution in surface and underground water bodies, as well as in soil. It comprises the rinsing and proper disposal or reuse of clean EPC. However, the rinsing transfers the hazard pesticides to water, which must be properly treated before discharged. In this work, we studied the application of a chemical oxidation reaction (Fenton process), based on the use of iron and hydrogen peroxide, enhanced by the use of solar radiation, at pilot plant scale in Cochabamba (Bolivia) for the removal of pesticides. The results demonstrated that solar photo-Fenton with low Fe ^2+^ concentrations is an effective method to remove dimethoate and atrazine. In conditions close to real applications, it achieved a removal over 99% of both pesticides after a solar irradiation period of 60 minutes. Further studies are needed for process optimization and for its coupling with biological or other post-treatments for the removal of transformation products.

## Introduction

Since the Green Revolution began in the 1960s, the use of pesticides has increasingly become essential to ensure the quantity and quality of food required to satisfy the growing human population. Pesticides are used to prevent, destroy, and control pests in order to secure agricultural crop production. However, the use of pesticides has always involved risks to human and environmental safety, as these compounds have the potential to cause adverse effects to non-target organisms ^1– 3^. Some of the most common unwanted sources of exposure to these chemicals are water resources impaired with pesticide residues, which typically reach the aquatic environment via runoff, soil leaching and spray drifting after its application ^4^.

Bolivia is a developing country with a cultivated area of more than 2,094,035 ha, whose main activity and economical income is agricultural production. The specific annual consumption of pesticides in conventional agriculture in Bolivia is among the highest in South America, registering a total legal amount of 33,464,329.94 kg (solid) and 156,752,721.1 L (liquid) of pesticides between 2010 and 2016 ^5^. According to Bascopé and colleagues ^6^, 72% of the 229 registered pesticides in Bolivia are toxic. From them, at least 78 are highly dangerous, 105 are prohibited in other countries and 75 are not authorized in the European Union ^1^. In particular, herbicides considered hazardous like atrazine (ATZ), glyphosate and paraquat are used in corn crops. Other dangerous insecticides like dimethoate (DIM) and chlorpyrifos are used in soybean and corn crops for the control of diverse plagues ^5^. All these products are bought, stored and used around the country, in many cases by uninformed, undertrained farmers ^7^ that are not totally aware of the dangers of these substances and make irrational and excessive use of pesticides ^8^.

Herbicide ATZ and pesticide DIM are used in high quantities in Bolivia, despite the fact they are considered highly hazardous pesticides by Pesticide Action Network International ^9^. ATZ has been found in surface and drinking water sources in rural areas of Cochabamba (Bolivia) at concentrations between 0.044 to 4.87 µg L ^-1^, and also in sediment in a range of 0.05 to 6.87 mg kg ^-1^ ^10^.******

Apart from the direct impact of the application of this massive amount of pesticides, Bolivian farmers’ misinformation leads to the accumulation of large quantities of empty pesticide containers (EPCs) on the margin of fields and rivers ^11^. This is another important source of environmental contamination ^8^, due to the natural wash-out of those receptacles or vaporization of the remaining content. The misuse of EPCs as water or food vessels has also been spotted ^6^ despite the dramatic danger it entails for human health, though this practice is marginal compared to other regions ^7^.

The Agricultural Inputs Suppliers Association ( *Asociación de Proveedores de Insumos Agropecuarios APIA*) in Bolivia carries out a Corporate Social Responsibility Program ( *Campo Limpio*) since 2005 to date as part of its environmental commitment. This program is oriented to the handling, final disposal, and recycling of containers of pesticides used in agriculture. In addition, some progress is being made towards adequate legislation for the integral management of EPCs, guaranteeing their environmentally adequate final disposal. However, currently there is a lack of information regarding the amount of poorly managed EPCs in Bolivia. Despite this, data from other world regions may help in getting a rough estimation. For instance, works performed in the Pella prefecture, Greece, reported 0.9 to 35.3 EPCs abandoned/poorly managed per hectare, depending on the crop type. In weight of pesticides, this represents 0.97 kg ha ^-1^ and 4.36 kg (farmer year) ^-1^ ^12^. Since in Bolivia the use of pesticides is less controlled than in the EU, considerably higher numbers could be expected ^3^; therefore, it seems clear that more efforts will be needed to reduce the presence of uncontrolled EPCs.

According to the Food and Agriculture Organization (FAO), safe management of EPCs requires that, before they are properly discarded to landfill, plastic recycling system or similar ^13^, the receptacles should be triple rinsed and taken out of service by punching or cutting to avoid their reuse as a vessel ^7, 14^. It is considered that the triple rinse, if done just after emptying the receptacle, avoiding pesticide cake formation ^15^ and dumping the rinsing water over the crop, removes potential pesticide exposures to human health and ecosystems by reducing the risk of contaminating soil, surface water, and groundwater ^13^. However, this cleansing transfers the pollution to a liquid phase that must be properly managed.

Some promising technologies for rinse wastewater treatment are light-driven Advanced Oxidation Processes (AOP), such as photo-Fenton. The light enhances in this case the production of highly reactive oxidant radical species, mainly hydroxyl radical (•OH) ^16^. The use of solar light, whose emission spectrum partially overlaps with the UV absorption spectrum of the Fenton reagent, enhances the performance of the process inexpensively. Moreover, its implementation is particularly interesting in those regions where the access to energy and technology are compromised. This is the case of Bolivia, where the high irradiation indexes (that doubles those obtained in middle latitudes and altitudes near sea level ^17^) make the solar photo-Fenton a potential treatment technology for rinse water polluted with pesticides in this region. However, as iron is one of the main reactants in the photo-Fenton process, it must be taken into account that, according to Bolivian legislation ^4^, the maximum concentration of this metal allowed to be discharged to water collection systems is 1 mg L ^-1^.

In accordance to this, the main aim of this work is to determine the viability of using solar photo-Fenton to treat EPC rinse wastewater, using DIM and ATZ as model pesticides. For the first time in Bolivia, the study has been carried out in a pilot plant placed in Cochabamba, a city characterized by its equatorial latitude and an altitude of 2570 meters above sea level (masl). Specific test conditions employed included the use of the pesticide’s commercial formulations and very low Fe ^2+^ dosage in river water matrix. The study was carried out in the framework of the EU project KNOWPEC (Knowledge for pesticides control/H2020-MSCA-RISE-2015).

## Methods

### Chemicals and river water matrix

Commercial ATZ (Atraxin 50% SC) was purchased from Shandong Qiaochang Chemical Co. Ltd. (China), and commercial DIM (Danadim Progress, 400EC) was purchased from CHEMINOVA A/S (Denmark). Other reactants: hydrogen peroxide (H _2_O _2_ 30% m/v, MERCK), iron sulphate heptahydrate (FeSO _4_.7H _2_O, MERCK, 99.5%), 1,10-phenanthroline, ammonium metavanadate, acetone and acetonitrile (Sigma Aldrich, reagent grade C _12_H _8_N _2_ · H _2_O, 98.5% NH _4_VO _3_, HPLC grade CH _3_COCH _3 _and CH _3_CN ), sodium bisulphite (NaHSO _3_, J.T. Baker). Pure ATZ (chromatography quality, Chem Service) and DIM (chromatography quality, Riedel-de Haen, 99.8%) were used for standards.

**Figure 1.**
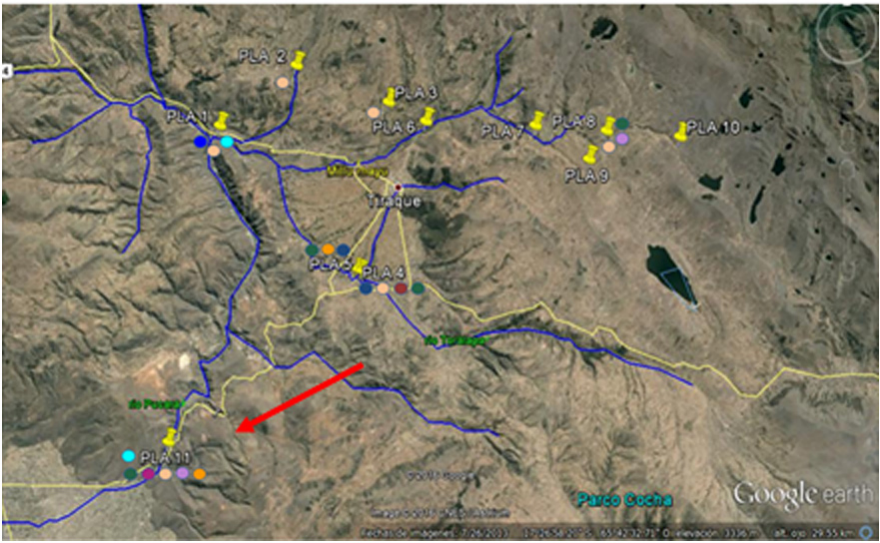
Pucara River and location of river water uptake PLA 11 (17°30'15.4"S 65°47'57.8"W). Maps Data: Google ©, 2016; CNES/Astrium; Recovery 02/09/2016.

Commercial pesticides were spiked to natural water collected from the Pucara river located in Punata (Cochabamba province) from point PLA 11 in Figure 1.

The water samples were collected through simple random sampling, with their respective sampling protocols and chain of custody. Samples for the analysis of pesticides were collected in one-liter amber glass bottles with a Teflon cap preserved with dichloromethane. Samples for the physicochemical parameters and degradation tests were kept in polyethylene bottles without preservative. They were transported to the C.A.S.A laboratory in refrigerated containers with dry ice. In the laboratory, the samples were stored in a cold chamber at 4°C prior to registration and recoded according to C.A.S.A standards.

The main quality parameters of river water at the sampling point, obtained according to the corresponding procedure of the standard methods ^18^, are detailed in Table 1.

**Table 1.** Quality parameters of Pucara river water and the standard method used ^18^. * Method: Environmental Protection Agency 8081A (adapted).

PARAMETER	Method	Detection limit	Units	CONCENTRATION
TOTAL SOLIDS	2540 B	0.001	mg L ^-1^	56.0
DISSOLVED SOLIDS	2540 C	0.001	mg L ^-1^	36.0
SUSPENDED SOLIDS	2540 D	0.001	mg L ^-1^	20.0
BICARBONATE	2320	0.01	mgCaCO3 L ^-1^	91.44
CARBONATE	2320B	0.01	mgCaCO3 L ^-1^	<0.01
TOTAL IRON	3500-Fe B	0.02	mgFe+3 L ^-1^	0.10
SULFATE	4500-SO4E	0.36	mgSO42- L ^-1^	35.17
ATRAZINE *		1.9	µg L ^-1^	6.8
DIMETHOATE *		0.03	µg L ^-1^	16.4

Experiments were conducted in Cochabamba, (17° 23' 33.7"South, 66° 8' 40" West, at 2570 masl), from 10 a.m. to 3 p.m., where the daily average solar radiation is between 5.4-5.7 kWh m ^-2^. Commercial ATZ and DIM were spiked in Pucara river water to simulate rinse effluents for this study. Experiments were carried out in a Solar DETOX ACADUS-2009/0.5 reactor with 15 L of capacity. Measures of global UV radiation in a range of 0 to 60.8 W m ^-2^ and wavelengths between 290 and 370 nm were obtained by means of an ACADUS 85-PLS radiometer, and the corresponding accumulated radiation energy Q (kJ L ^-1^) was calculated as described elsewhere ^19^. Two different types of simulated wastewaters were tested: one with 10 mg L ^-1^ DIM alone and another with a mixture of commercial DIM and ATZ, at a final concentration of 10 mg L ^-1^ each. The pH was kept between 2.8 to 3.2 along all the reactions. Several experiments were carried out adding different amounts of H _2_O _2_ (from 50 to 200 mg L ^-1^) and low concentrations of FeSO _4_·7H _2_O (from 0.3 to 1.5 mg L ^-1^ of Fe ^+2^), in accordance with the discharge limits of the regional legislation ^20^. Photodegradation experiments without the addition of H _2_O _2 _were also performed. Pesticide degradation was monitored by taking samples at different time intervals until the complete elimination of pesticides.

### Analytical procedures for the pesticide degradation test

Pesticides were extracted from the water samples by liquid extraction with dichloromethane, according to the Environmental Protection Agency 3510 method. Then the extracts were cleaned and concentrated in a rotary evaporator. A second concentration step was carried out by applying a nitrogen flow and using acetonitrile as recovering solvent for injection into the chromatograph. Quantitation was performed by a high-performance liquid chromatograph using an Agilent 1100 equipped with a diode array detector (DAD) and an autosampler. The employed column was a Hypersil ODS 4x125 mm of 5 µm particle diameter and the software ChemStation, version for LC-3D Rev.A.10.02(1757). The mobile phase consisted of 60:40 volumetric mixtures of Milli Q water and acetonitrile, and the flowrate was kept at 1 mL min ^-1^. The injection volume was 60 μL. The detection wavelength was 200 nm for DIM and 224 nm for ATZ. Samples were previously filtrated with 0.45 µm PVDF filters. The concentration of dissolved iron and hydrogen peroxide was determined spectrophotometrically through the phenanthroline and metavanadate methods, respectively ^21^.

## Results and discussion

### Performance of Photo-Fenton on DIM

First, dark controls for DIM and ATZ solutions were performed for 6h. Results revealed that neither hydrolysis nor volatilization occurred significantly during the exposure period (DIM concentration diminished less than 3.2% at pH=2.7), which indicates that the observed decreases in pesticide concentrations during photodegradation experiments were mainly caused by photochemical processes.

**Figure 2.**
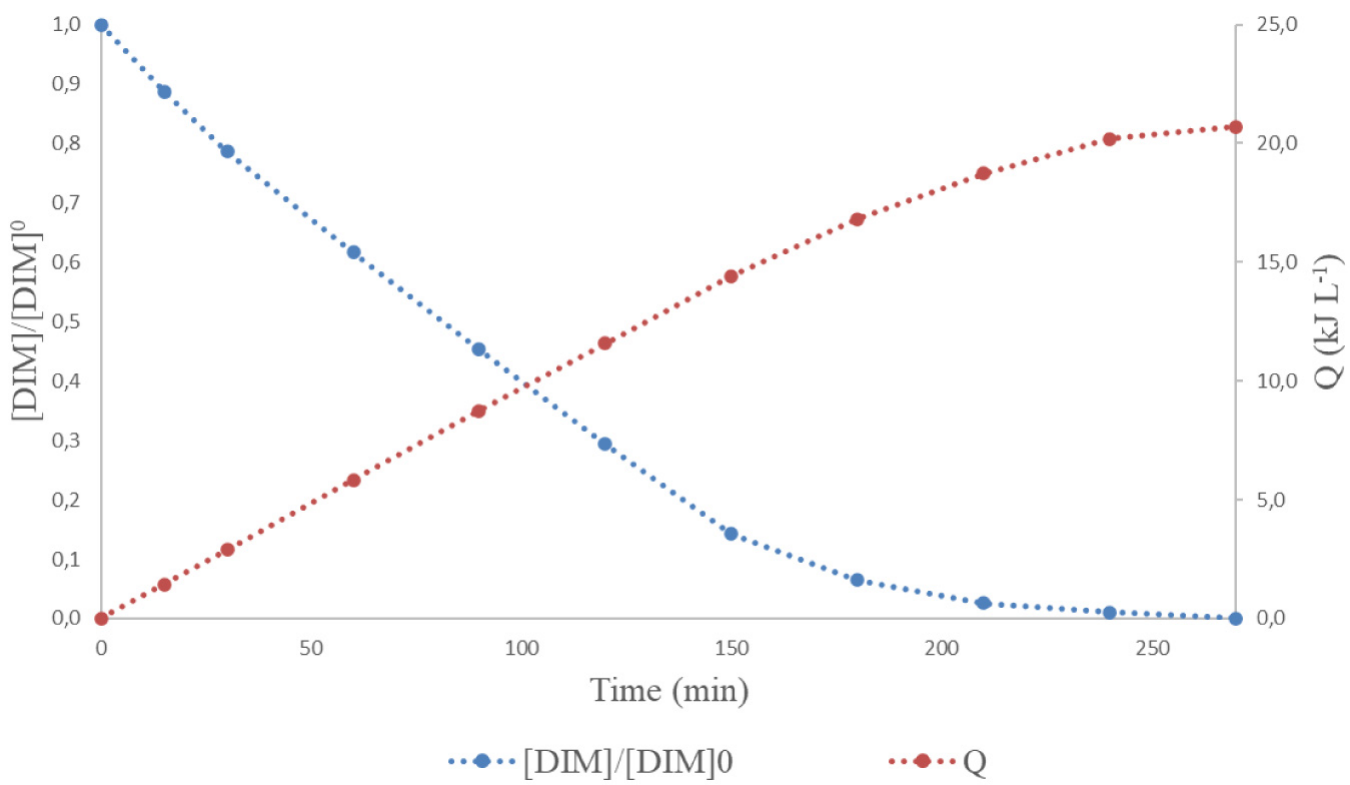
Normalized dimethoate (DIM) concentration and accumulated energy, Q, along reaction time. (pH= 3.2). [Dim] _0 _= 10 mg L ^-1^; [Fe] _0 _= 1 mg L ^-1^; [H _2_O _2_] _0 _= 0 mg L ^-1^).

Figure 2 shows the normalized DIM concentration and accumulated solar energy with time, in the presence of 1 mg L ^-1^ of dissolved Fe ^2+^ and pH= 3.2, in the absence of hydrogen peroxide for the experiments with this pesticide as the only target compound _._

As can be observed in Figure 2, photodegradation occurs without the addition of H _2_O _2_. This photodegradation reached an extent of 54% removal after 90 minutes of exposure to radiation (8.8 kJ·L ^-1^ of accumulated energy) and continued until the total decomposition of DIM load after 210 minutes (corresponding to 18.7 kJ·L ^-1^ of accumulated energy). Direct photolysis (i.e., photocleavage) of DIM is unlikely to occur due to the small portion of the sun emission spectrum corresponding to wavelengths below 300 nm, together with the low absorbance of this compound at wavelengths above 300 nm. Instead, indirect photoreactions may constitute the principal mechanisms by which the target compound is degraded. It is well demonstrated that dissolved organic matter (DOM) can indirectly induce phototransformations in natural waters. On one hand, sunlight induced excited DOM triplet states ( ^3^DOM*) may undergo photoionization by electron transfer to DOM molecules, which could in turn become transformed ^22^. Moreover, ^3^DOM* is considered to be the main precursor of singlet oxygen ( ^1^O _2_) in natural waters by quenching with dissolved molecular oxygen, promoting generation of other reactive oxygen species (ROS). On the other hand, however, phenolic antioxidant moieties of humic acids from DOM may inhibit the excited triplet-induced oxidation. The presence of inorganic compounds such as carbonate, sulphate or chloride may also contribute to or inhibit the indirect photodegradation of contaminants ^22^. Taking all this into account, the effectiveness of sunlight irradiation on DIM depletion relies most probably on the photosensitizing capability of the natural organic matter present in Pucara river, as suggested by the high value of dissolved solids analysed (36 mgL ^-1^). However, the contribution of other possible indirect phototransformations should not be discarded, as observed with other organophosphorus pesticides such as dichlorvos ^23^. Upon light absorption, electron transfer to dissolved oxygen and subsequently a superoxide-mediated mechanism appears to control the sunlight photodegradation of this pesticide. Superoxide dismutation generates hydrogen peroxide, which upon irradiation forms •OH, which is mainly responsible for DIM degradation in the studied system. The mentioned mechanism is especially effective under acidic conditions such as those employed in the experiment presented in Figure 2. Considering the high depletion rate observed for DIM under sunlight irradiation in the studied conditions, the possible contribution of other indirect photo-transformation pathways should be further evaluated.

Addition of hydrogen peroxide to the reaction mixture was aimed at increasing the rate of transformation of DIM by taking advantage of its combination with iron and solar irradiation, which enables the photo-Fenton process to take place. However, Bolivian regulations concerning iron in discharged waters forced the use of low concentrations of this catalyst in this study, namely 1 mg L ^-1 ^at most. Under these conditions, using typical Fe/H _2_O _2_ ratios (i.e., between 0.1 and 0.2 on the basis of mass) resulted in poor performances of the process regarding target pollutant degradation, with no significant improvements achieved compared to the experiment conducted in the absence of hydrogen peroxide. This being so, the oxidant concentration was fixed at 200 mg L ^-1^. For this condition, significant improvements in removal efficiencies were observed compared to the control test.

**Figure 3.**
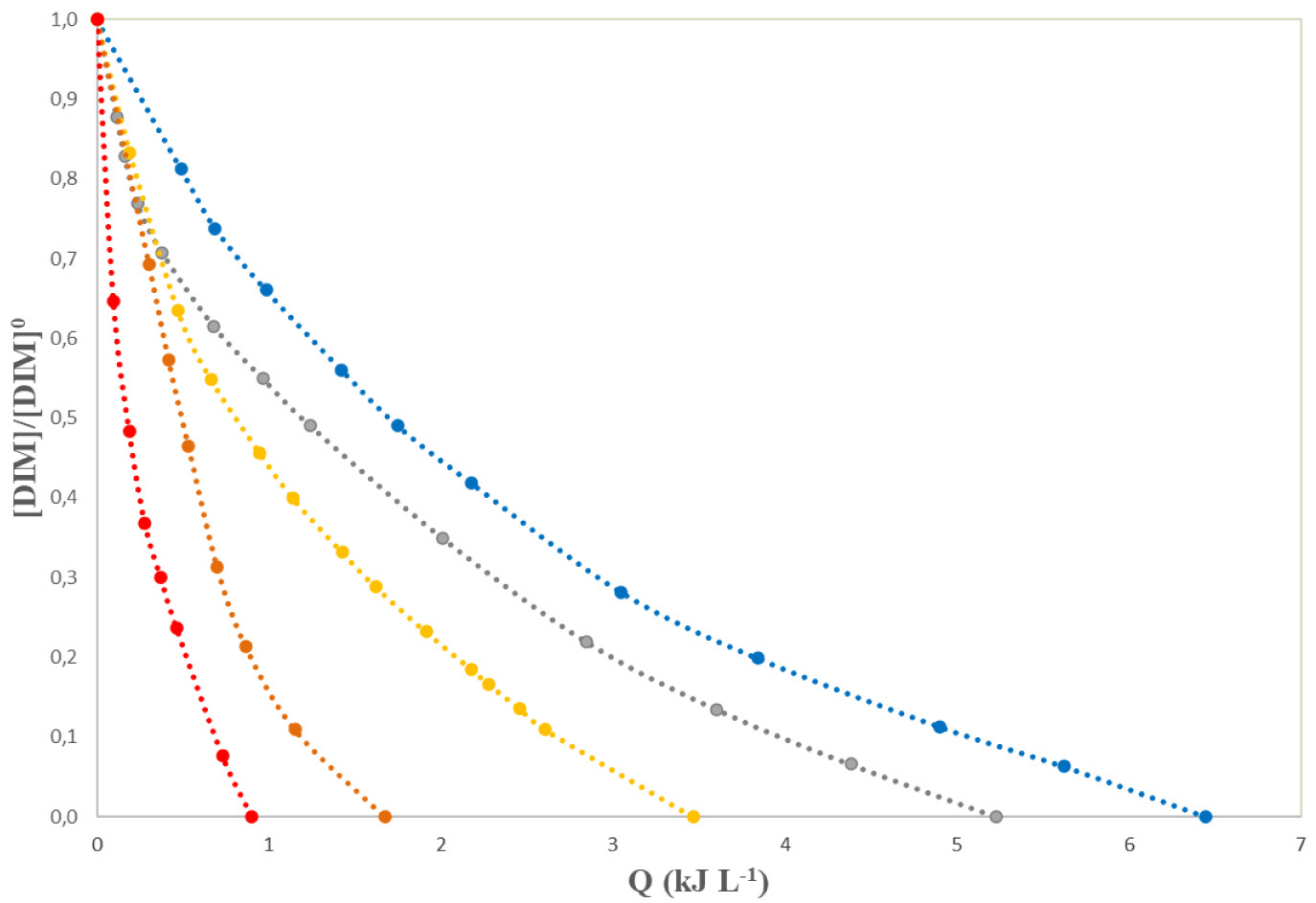
Normalized dimethoate (DIM) concentration versus accumulated energy, Q, for experiments with [H _2_O _2_] _0_=200 mg L ^-1^ and different [Fe ^2+^], blue dots: 0.3 mg L ^-1^; grey dots: 0.5 mg L ^-1^; yellow dots: 0.7 mg L ^-1^; orange dots: 1.0 mg L ^-1^; red dots: 1.5 mg L ^-1^.

Figure 3 compares the DIM degradation in the presence of 200 mg·L ^-1^ of hydrogen peroxide with different concentrations of ferrous ion (between 1.5 to 0.5 mg L ^-1^) versus the accumulated energy. In every case, all DIM load is depleted. Furthermore, it can be seen that the peroxide is able to noticeably decrease the amount of energy required for the total degradation rate of the target compound compared to the photodegradation or blank experiments. By doubling ferrous ion content from 0.5 to 1.0 mgL ^-1^, 55% degradation of the DIM load was achieved with almost half of the accumulated energy (0.53 and 0.97 kJ L ^-1^, respectively). That beneficial effect was also observed when Fe ^2+^ concentration increases from 1.0 to 1.5 (51% of degradation at only 0.19 kJ L ^-1^ of accumulated energy for the experiment with 1.5 mg L ^-1^ of Fe ^2+^). The catalytic effect of iron is clearly reflected when comparing total DIM degradation and reaction times, which were 10, 40 and 90 minutes for experiments with 1.5, 1 and 0.5 mg L ^-1^ of Fe ^2+^, respectively. Iron content in solution remained unchanged during all the experiments, as the solution’s pH value was kept at acidic values throughout the reaction time (around pH=2.8).

**Figure 4.**
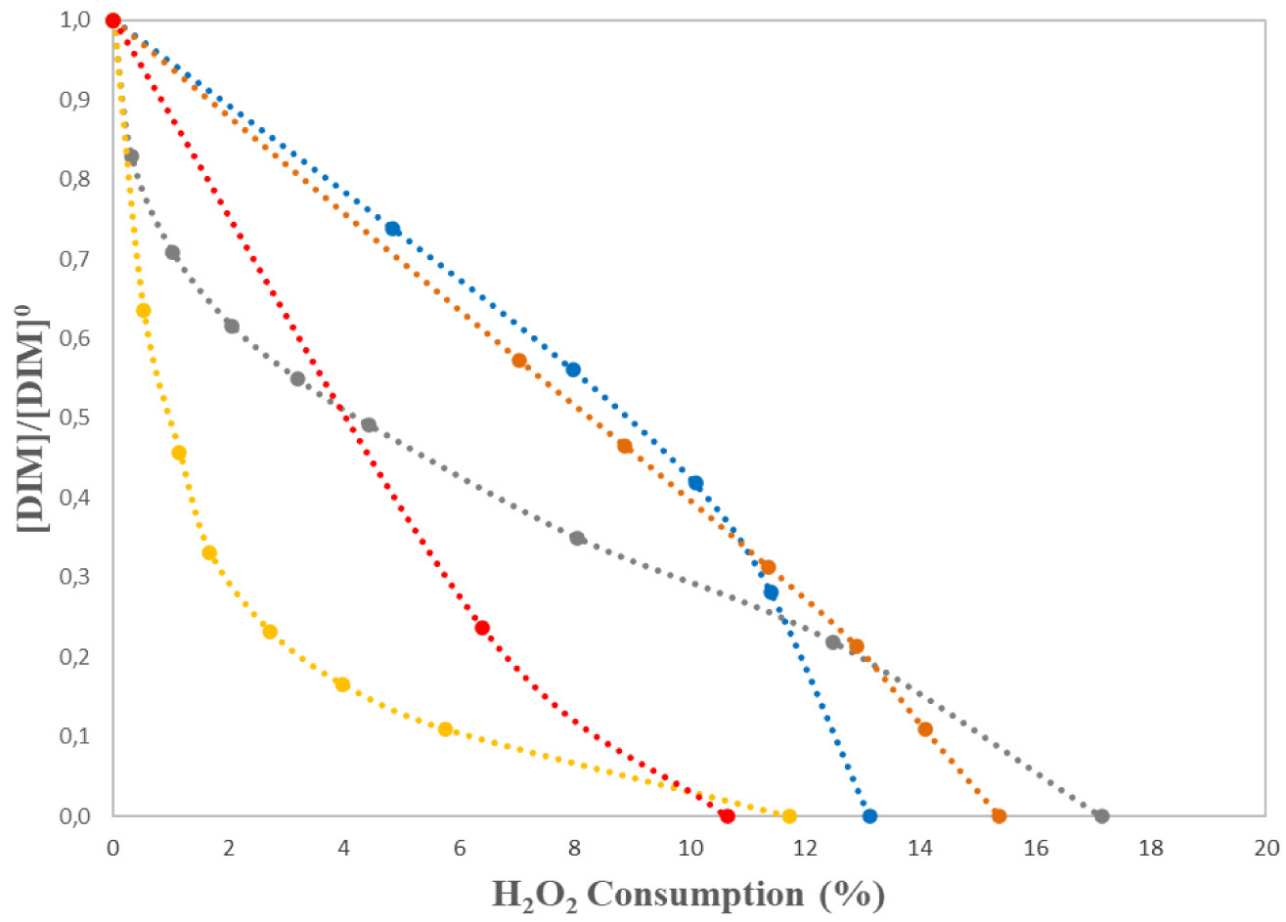
Normalized dimethoate (DIM) concentration versus hydrogen peroxide consumption [H _2_O _2_] _0_=200 mg L ^-1 ^and different [Fe ^2+^], blue dots: 0.3 mg L ^-1^; grey dots: 0.5 mg L ^-1^; yellow dots: 0.7 mg L ^-1^; orange dots: 1.0 mg L ^-1^; red dots: 1.5 mg L ^-1^.

According to the observed trend, higher values of Fe ^2+^ concentration would probably show better results, allowing a reduction of the amount of hydrogen peroxide required to obtain acceptable treatment performances. Two other experiments were performed in order to confirm this premise. One experiment was conducted with 50 mg L ^-1^ of H _2_O _2_ and 10 mg L ^-1^ of Fe ^2+^. In this experiment, DIM could be completely removed in less than a minute. On the contrary, in another experiment employing the same catalyst/oxidant ratio (i.e., 0.2) but half the amount of reagents (5 and 25 mg L ^-1^ of Fe ^2+^ and H _2_O _2_, respectively) the observed removal performance dropped dramatically, with an accumulated energy consumption of 5.27 kJL ^-1^, similarly to the experiment with 0.5 mg L ^-1^ of Fe ^2+^ in Figure 3. This low performance could be attributed to the hydroxyl radical scavenging effect exerted by some of the typical components of natural water, mainly organic and inorganic carbon species. From these results several conclusions can be drawn: on one hand, the rate of pesticide removal during the process is dramatically limited by the low amount of iron permitted to be used by Bolivian legislation in this particular application. On the other, under low catalyst concentration, hydrogen peroxide should be added in larger excess ratios than those typically employed (e.g., iron/hydrogen peroxide ratios of about 0.005) due to the high natural organic matter and inorganic salts content of Pucara river. However, the fact that less than 20% of the initial hydrogen peroxide content is decomposed in treatments using initial concentrations of this reagent of 200 mg L ^-1^, as shown in Figure 4, seems to indicate that optimized initial Fe ^2+ ^and H _2_O _2_ concentration could be used in a real application. In that case, post-treatments like iron precipitation and sludge separation should be required to enforce iron limitations.

**Figure 5.**
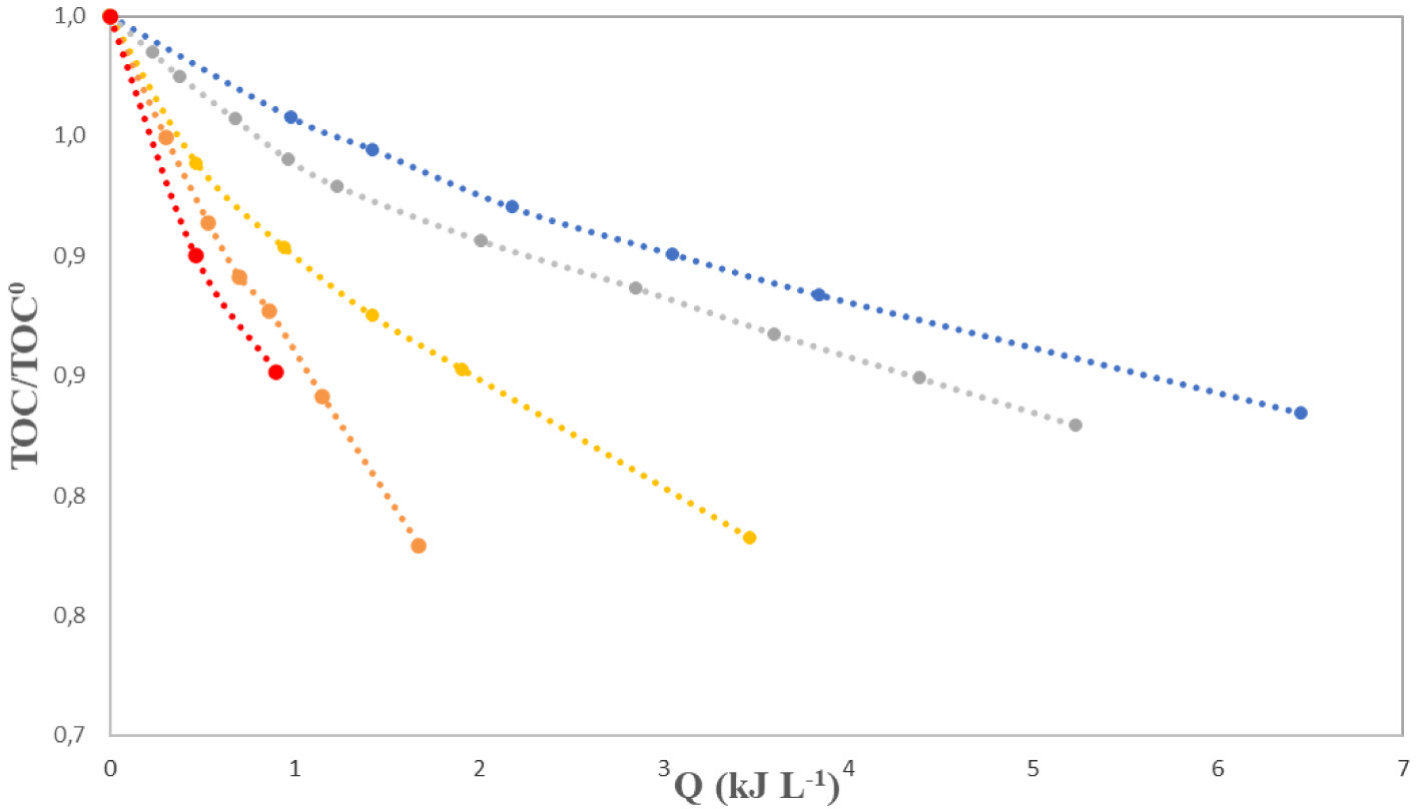
Normalized total organic carbon (TOC) content versus accumulated energy, Q, for experiments with [H _2_O _2_] _0_=200 mg L ^-1^ and different [Fe ^2+^], blue dots: 0.3 mg L ^-1^; grey dots: 0.5 mg L ^-1^; yellow dots: 0.7 mg L ^-1^; orange dots: 1.0 mg L ^-1^; red dots: 1.5 mg L ^-1^.

Applied doses of chemical oxidants, catalysts and radiation employed in oxidation processes, such as the solar photo-Fenton applied in this study, are far from achieving the complete mineralization of organic pollutants and natural organic matter contained in the samples. Instead, transformation products are formed. This is illustrated by the total organic carbon (TOC) evolution depicted in Figure 5, which revealed limited reductions of this parameter, between just 10 and 23%, for the conditions applied in the different experiments. In general, faster degradations were obtained with increasing concentrations of iron, particularly at 1 and 1.5 mg L ^-1^. Under these two different experimental conditions, in fact, only small differences were observed. Therefore, the maximum iron level permitted by the current Bolivian legislation would be enough to achieve mineralization levels like those obtained using a higher catalyst concentration. In addition, the observed trends indicate that only the extension of the irradiation time under these treatment scenarios would allow a higher mineralization grade to be reached. Some optimization may, however, be conducted in this sense to reach, to the extent possible, the most economical and convenient set of operational conditions. In any case, two details merit mention at this point. On one hand, TOC measurements accounted for both the target pesticides, their generated intermediates, and the organic matter naturally present in the river water matrix. This means that a significant part of the reactive oxidant species formed are consumed in degrading natural organic carbon. On the other, previous experimental evidence shows that primary or secondary attack of oxidants such as •OH on target pesticides leads to proportional reductions of their initial toxicity ^24^. Thus, the latter is in principle likely to be reduced upon oxidative treatment of DIM-containing waters under the tested conditions.

### Performance of photo-Fenton on the mixture DIM-ATZ

**Figure 6.**
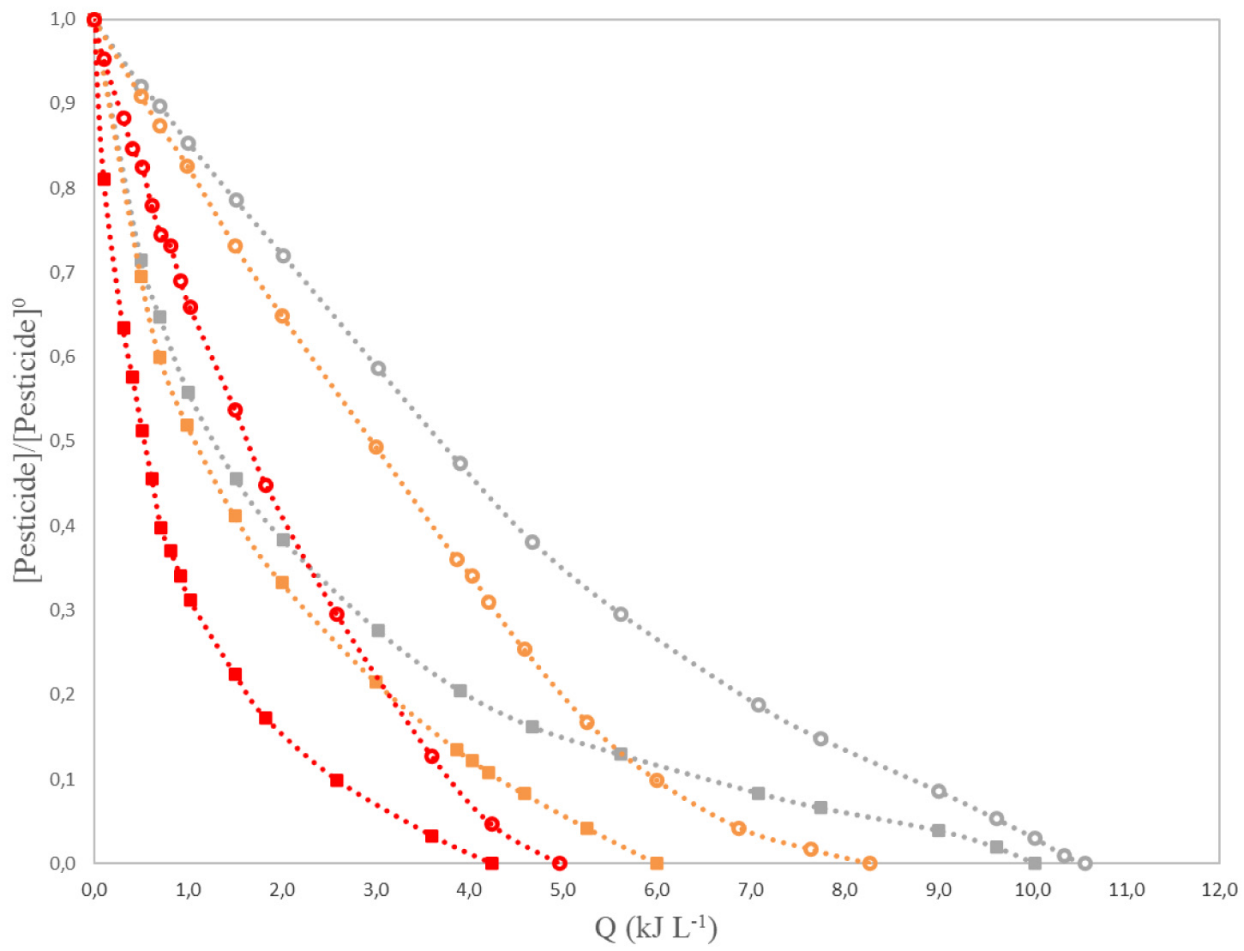
Normalized dimethoate (circles) and atrazine (squares) concentration versus accumulated energy, Q. [DIM] _0_=10 mg L ^-1^; [ATZ] _0_=10 mg L ^-1^; [H _2_O _2_] _0_=200 mg L ^-1 ^and different [Fe ^2+^], grey dots: 0.5 mg L ^-1^; orange dots: 1.0 mg L ^-1^; red dots: 1.5 mg L ^-1^.

**Figure 7.**
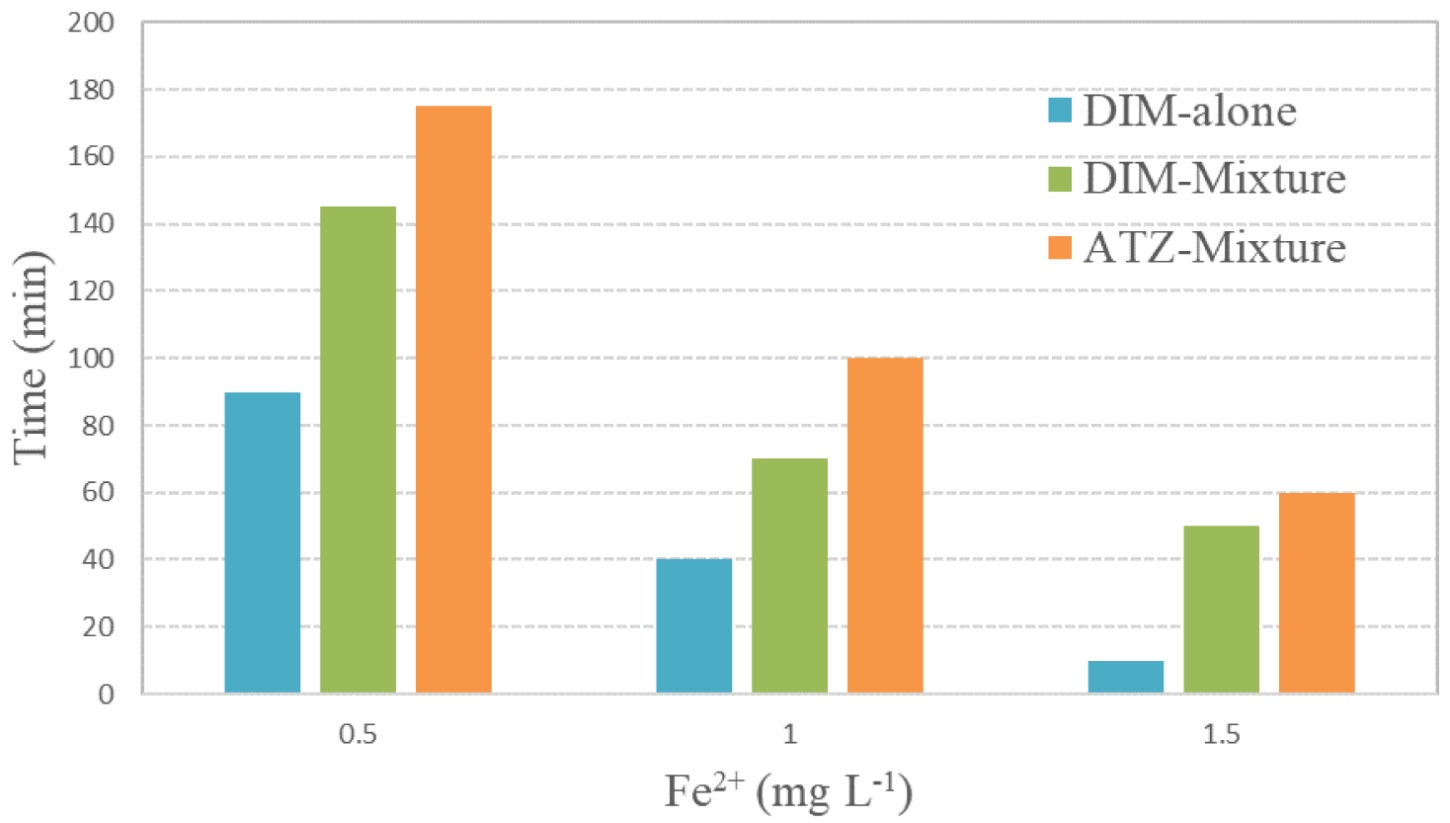
Solar irradiation time required for total removal of dimethoate (DIM, alone and in the mixture) and atrazine (ATZ, mixture) versus different [Fe ^2+^]. [DIM] _0_=10 mg L ^-1^; [ATZ] _0_=10 mg L ^-1^; [H _2_O _2_] ] _0_=200 mg L ^-1^.

A mixture of different pesticide formulations can easily compose rinse waters coming from EPCs. In an attempt to simulate this potential scenario, mixtures of DIM and ATZ were prepared in river water and treated by photo-Fenton under previously tested operational conditions. As observed in Figure 6, tested operational conditions also achieved total herbicide and pesticide depletion. However, those degradations required longer irradiation times compared to experiments with only one target pesticide and therefore higher accumulated energy values, as it is depicted in Figure 7. In addition, differences in the oxidation rates of both compounds are clear, ATZ always being slower in its degradation than DIM. ATZ has a second-order rate constant for its reaction with hydroxyl radical of 3·10 ^9^ M ^-1 ^s ^-1^ ^25^, whereas this information is not available, to the best of our knowledge, in the case of DIM. However, a higher value compared to that in the case of DIM would explain the observed differences. Alternatively, it is possible that ATZ, a triazine compound with a completely different structure than that of DIM, was less prone to undergo direct or indirect photoreactions. In this case, the contribution of this particular mechanism to the overall observed degradation would be reduced.

**Figure 8.**
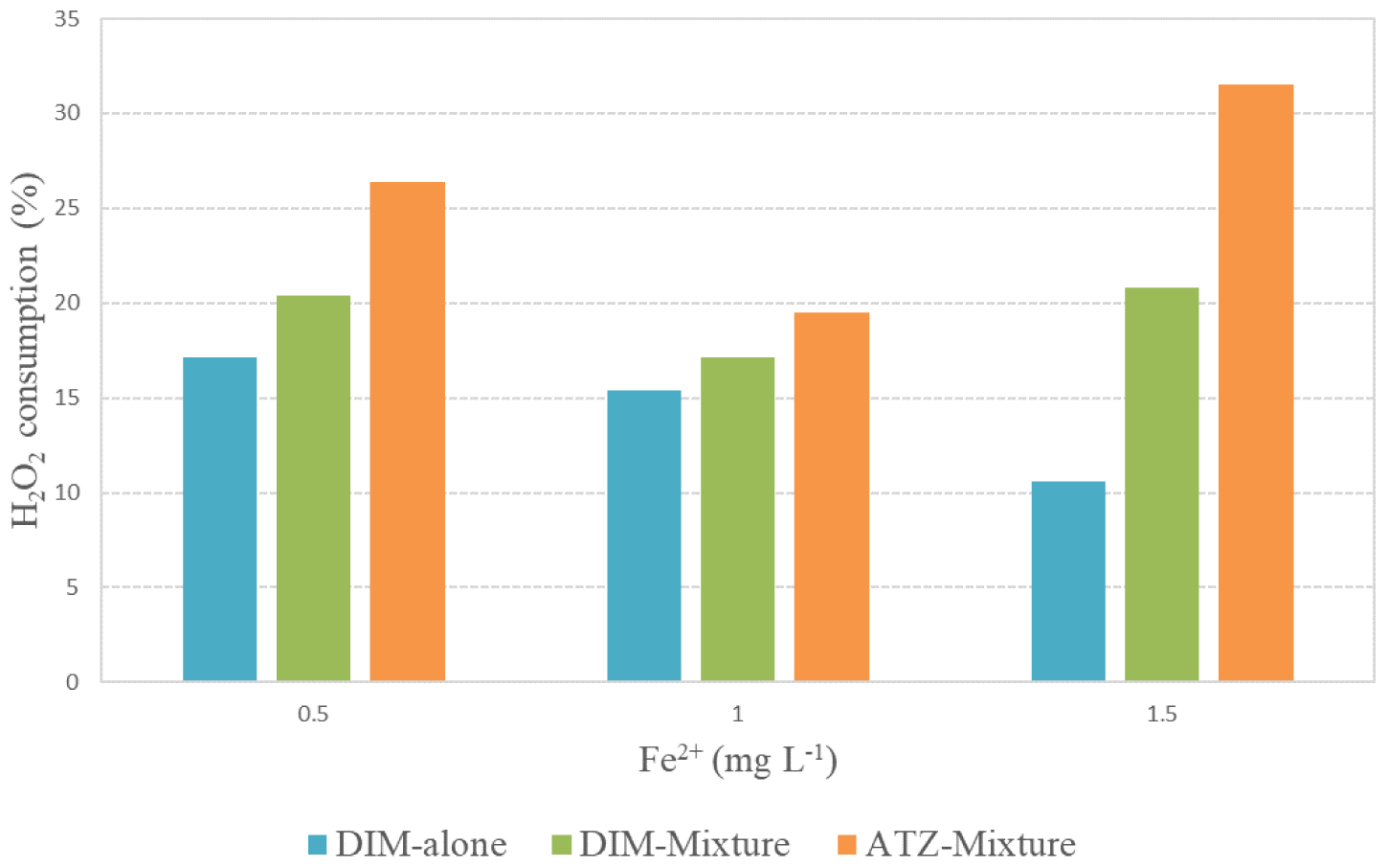
Percentage of H _2_O _2_ consumption for total removal of dimethoate (DIM, alone and in the mixture) and atrazine (ATZ, mixture) versus different [Fe ^2+^]. [DIM] _0_=10 mg L ^-1^; [ATZ] _0_=10 mg L ^-1^; [H _2_O _2_] ] _0_=200 mg L ^-1^.

Regarding oxidant consumption during the experiment, hydrogen peroxide reduction for total depletion of pesticides in the mixture was significantly higher compared to the single target test; up to 31.5% and 10.6%, respectively, for the experiment with 1.5 mg L ^-1^ of Fe ^2+^, summarized in Figure 8. This is justified by the presence of higher pesticide concentration in the mixture, which consequently implies a higher organic matter content. Moreover, higher peroxide consumption in the test performed with 1.5 mg L ^-1^ of Fe ^2+^ was clearly observed compared with lower iron concentration experiments.

**Figure 9.**
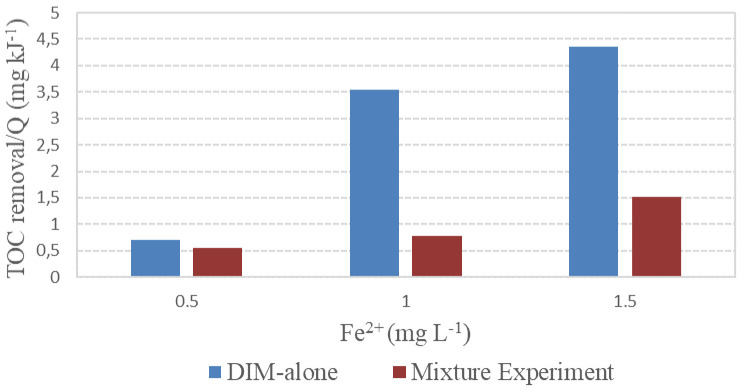
Total organic carbon (TOC) removal per accumulated energy applied for the dimethoate (DIM) alone experiment and for the mixture of dimethoate and atrazine experiment versus different [Fe ^2+^]. [DIM] _0_=10 mg L ^-1^; [ATZ] _0_=10 mg L ^-1^; [H _2_O _2_] ] _0_=200 mg L ^-1^.

During the degradation of the pesticide mixture, TOC had a similar behaviour compared to the individual degradation of DIM, requiring less accumulated energy at a higher concentration of Fe ^+2^. However, the mixture experiment required a greater amount of accumulated energy for its partial mineralization, particularly at higher Fe ^+2^ doses tested. This can be observed in Figure 9, where the ratio TOC-removal/accumulated energy is represented against the iron dose applied. Again, the higher pesticide content in the mixture would have competed for the hydroxyl radicals produced throughout the irradiation process, decreasing the mineralization efficiency of the photo-Fenton process. These results point out the convenience of biological post-treatment for the proper disposal of the oxidized effluent.

## Conclusions

This work demonstrates that solar powered photo-Fenton is a suitable technology for depolluting water streams coming from the EPC rinsing process, even at very low ferrous iron doses (below 1.5 mg L ^-1^). When using 200 mg L ^-1 ^of hydrogen peroxide, the process was able to completely deplete DIM and ATZ mixtures at an initial concentration of 10 mg L ^-1 ^each, both in its commercial formulation and in a river water matrix. The large irradiation times and oxidant doses required for obtaining acceptable levels of pesticide removal can be explained on the basis of the low iron doses employed (taking into consideration legal discharge limits in Bolivia) and the relatively high content of both organic and inorganic carbon compounds in the water matrix, which exert a significant scavenging effect over ROS, responsible for pollutant oxidation. However, due to this, total pesticide removal is accompanied with low oxidant consumption and TOC mineralization. The photo-Fenton process has room for improvement in tuning its operation parameters, which would entail lower hydrogen peroxide and accumulated energy requirements if a biological post-treatment is considered, or the application of higher values of ferrous ion doses. In that case, a posterior precipitation step could be considered for lowering iron content and enforcing discharge limits.

## Data availability

Repositori de dades de recerca: Solar pilot-plant results of photo-Fenton process treating water polluted with pesticides in Bolivia. https://doi.org/10.34810/data109 ^26^.

This project contains the following underlying data:

- RawData_ Dimethoate.xlsx
- RawData_Mixture.xlsx

Data are available under the terms of the Creative Commons Attribution 4.0 International license (CC-BY 4.0).
