# Supplementary material for: Application of solar-based oxidation to the management of empty pesticide container rinse water in Bolivia
Source: Open Res Eur. 2021 Jun 18;1:70. [Version 1] doi: 10.12688/openreseurope.13555.1 (PMC10445800; doi:10.12688/openreseurope.13555.1)
Supplement: Peer review history

# Peer review: Reviewer response for version 1

**DOI:** 10.21956/openreseurope.14625.r27615

**Published:** 2021-09-16

**Authors:** Yuan xiangjuan

**Affiliations:** 1 School of Environmental Engineering, Wuhan Textile University, Wuhan, China

The authors investigated the solar photo-Fenton process for the treatment of empty pesticide containers rinsing water. The results exhibited that the solar photo-Fenton with low Fe2+ dosage could achieve efficient dimethoate and atrazine removal, providing an effective alternative process for pesticide wastewater treament. This is an interesting, significant and applicable study for the elimination of different pesticides in surface water and rinsing water. 

 The authors should work on the following specific comments to improve the reports.

1. The data represented in the Figures should have the standard deviations. And the legends should also be included in the figures, making the data more intuitive.
2. The average decomposition of H2O2 was about 10%~20%. There are amounts of H2O2 residual in the effluent. It's better to introduce some post-treatments for the proper disposal of the massive oxidants in the effluent, which could make the solar photo-Fenton more applicable.

Is the study design appropriate and does the work have academic merit?

Yes

Is the work clearly and accurately presented and does it cite the current literature?

Yes

If applicable, is the statistical analysis and its interpretation appropriate?

Yes

Are all the source data underlying the results available to ensure full reproducibility?

Partly

Are the conclusions drawn adequately supported by the results?

Yes

Are sufficient details of methods and analysis provided to allow replication by others?

Yes

Reviewer Expertise:

The paper included some results of wastewater treatment, advanced oxidation processes, and pesticides abatement, which are within my area of research.

I confirm that I have read this submission and believe that I have an appropriate level of expertise to confirm that it is of an acceptable scientific standard.

# Peer review: Reviewer response for version 1

**DOI:** 10.21956/openreseurope.14625.r27173

**Published:** 2021-07-08

**Authors:** Rui Martins

**Affiliations:** 1 University of Coimbra, CIEPQPF – Chemical Engineering Processes and Forest Products Research Center, Department of Chemical Engineering, Faculty of Sciences and Technology, University of Coimbra, Coimbra, Portugal

This is a very interesting paper dealing with an important environmental problem related with the pesticides water pollution. Particularly the disposal of empty pesticides containers is an environmental risk. The management strategy, involving these receptacles triple rinsing before disposal, transfers the problem to the water. Solar photo-Fenton is indeed an interesting approach to treat that contaminated water. Moreover, the use of free solar energy is an important advantage making the process low cost. Besides, the authors proved that the use of low iron concentrations (bearing in mind the legislation regarding water iron content for discharge) may be used with high abatement of the pesticides. Thus this is a very important contribution showing a low cost technology for the abatement of these cumbersome pollutants.

 Authors should only address the following minor aspects:

- Some information should be given regarding the reproducibility of the results. Standard deviations should be given. 
- It would be helpful for the reader to have the molecular structure of the pesticides analyzed that way the authors statement regarding the fact that ATZ has a molecular structure making it "less prone to undergo direct or indirect photo reactions." would be more understandable.

Is the study design appropriate and does the work have academic merit?

Yes

Is the work clearly and accurately presented and does it cite the current literature?

Yes

If applicable, is the statistical analysis and its interpretation appropriate?

Partly

Are all the source data underlying the results available to ensure full reproducibility?

Yes

Are the conclusions drawn adequately supported by the results?

Yes

Are sufficient details of methods and analysis provided to allow replication by others?

Yes

Reviewer Expertise:

The paper is totally within my area of expertise: advanced oxidation processes for water contaminants removal.

I confirm that I have read this submission and believe that I have an appropriate level of expertise to confirm that it is of an acceptable scientific standard.
